# Uptake of Community-Based HIV Testing during a Multi-Disease Health Campaign in Rural Uganda

**DOI:** 10.1371/journal.pone.0084317

**Published:** 2014-01-02

**Authors:** Gabriel Chamie, Dalsone Kwarisiima, Tamara D. Clark, Jane Kabami, Vivek Jain, Elvin Geng, Laura B. Balzer, Maya L. Petersen, Harsha Thirumurthy, Edwin D. Charlebois, Moses R. Kamya, Diane V. Havlir

**Affiliations:** 1 HIV/AIDS Division, Department of Medicine, San Francisco General Hospital, University of California San Francisco, San Francisco, United States of America; 2 Makerere University-University of California San Francisco (MU-UCSF) Research Collaboration, Uganda; 3 The Sustainable East Africa Research in Community Health (SEARCH) Consortium; 4 Mulago-Mbarara Joint AIDS Program, Kampala and Mbarara, Uganda; 5 School of Public Health, University of California, Berkeley, United States of America; 6 Gillings School of Global Public Health, University of North Carolina at Chapel Hill, United States of America; 7 Center for AIDS Prevention Studies, Department of Medicine, University of California San Francisco, San Francisco, United States of America; 8 Department of Medicine, School of Medicine, Makerere University College of Health Sciences, Kampala, Uganda; Istituto Superiore di Sanità, Italy

## Abstract

**Background:**

The high burden of undiagnosed HIV in sub-Saharan Africa is a major obstacle for HIV prevention and treatment. Multi-disease, community health campaigns (CHCs) offering HIV testing are a successful approach to rapidly increase HIV testing rates and identify undiagnosed HIV. However, a greater understanding of population-level uptake is needed to maximize effectiveness of this approach.

**Methods:**

After community sensitization and a census, a five-day campaign was performed in May 2012 in a rural Ugandan community. The census enumerated all residents, capturing demographics, household location, and fingerprint biometrics. The CHC included point-of-care screening for HIV, malaria, TB, hypertension and diabetes. Residents who attended vs. did not attend the CHC were compared to determine predictors of participation.

**Results:**

Over 12 days, 18 census workers enumerated 6,343 residents. 501 additional residents were identified at the campaign, for a total community population of 6,844. 4,323 (63%) residents and 556 non-residents attended the campaign. HIV tests were performed in 4,795/4,879 (98.3%) participants; 1,836 (38%) reported no prior HIV testing. Of 2674 adults tested, 257 (10%) were HIV-infected; 125/257 (49%) reported newly diagnosed HIV. In unadjusted analyses, adult resident campaign non-participation was associated with male sex (62% male vs. 67% female participation, p = 0.003), younger median age (27 years in non-participants vs. 32 in participants; p<0.001), and marital status (48% single vs. 71% married/widowed/divorced participation; p<0.001). In multivariate analysis, single adults were significantly less likely to attend the campaign than non-single adults (relative risk [RR]: 0.63 [95% CI: 0.53–0.74]; p<0.001), and adults at home vs. not home during census activities were significantly more likely to attend the campaign (RR: 1.20 [95% CI: 1.13–1.28]; p<0.001).

**Conclusions:**

CHCs provide a rapid approach to testing a majority of residents for HIV in rural African settings. However, complementary strategies are still needed to engage young, single adults and achieve universal testing.

## Introduction

The high burden of undiagnosed HIV represents a major obstacle to implementation of HIV prevention and antiretroviral treatment (ART) strategies in sub-Saharan Africa. Late HIV diagnosis translates into missed opportunities for HIV prevention and delayed antiretroviral treatment resulting in increased HIV-related morbidity and mortality, and ongoing HIV transmission. In Uganda, less than 25% of HIV-infected persons are estimated to be aware of their status, and among 15–49 year olds one-third of women and over half of men have never tested for HIV [1], [2].

Health facility-based approaches to HIV testing in rural Africa are limited by several factors: cost of patient travel and waiting time when accessing centralized services, stigma, lack of awareness of HIV risk, and the minimal or non-specific symptoms experienced by many patients early in HIV disease [3], [4], [5]. Provider-initiated voluntary counseling and testing succeeds in reaching clinic patients who might not otherwise seek testing, but is dependent on a person seeking medical care [6], [7]. As a consequence, late presentations to HIV care are common. In a recent study, the median CD4 count at HIV diagnosis in an urban Ugandan hospital was <250 cells/μL [8].

Community health campaigns (CHC) that offer rapid HIV testing can close the “gap” between those who know and those who do not know their HIV status. CHCs aim to achieve universal testing across a community by removing significant barriers to HIV testing in a high-throughput format. CHCs offer HIV testing within broader service delivery (such as hypertension screening for adults and deworming of young children), thereby normalizing HIV testing as routine health care. CHCs also decentralize testing locations to minimize travel costs and waiting time, and actively mobilize community members to attend regardless of perceived risk. As a consequence, CHCs have succeeded in identifying large populations of persons reporting no prior testing, and in diagnosing HIV early [9], [10].

We have previously demonstrated high uptake of community-wide HIV testing with CHCs in rural Uganda [9]. In the present study, we implemented a census followed by a repeat five-day, high-throughput HIV testing and referral campaign in order to better understand barriers to CHC participation for residents of a rural Ugandan community. This information is critical as CHC approaches are scaled up to maximize their impact and to develop complementary testing strategies for nonparticipants.

## Methods

In May 2011, a multi-disease community health campaign (CHC) was conducted in a rural community, Kakyerere parish, in Mbarara District southwestern Uganda, with the results previously described [9]. In April and May 2012, an augmented study was conducted in the same parish, and included community sensitization, a baseline community census (to accurately measure participation rates and study reasons for non-participation), and a multi-disease CHC. We sought to understand predictors of participation in a CHC, a public-health approach to universal HIV testing.

### Community sensitization

In an effort to maximize campaign uptake, regional and village political leaders implemented community sensitization activities one month before the campaign. Routine meetings were held between the investigators and the village leaders to answer questions raised by community members and to emphasize that the campaign services were available to all community residents regardless of HIV status, age, sex, or prior engagement in clinical care. Sensitization activities included announcements at local places of worship and at village gatherings as chosen by village leaders (including weddings and funerals), and distribution of colorful posters and flyers describing the campaign services provided written in both English and Runyankole (the local language). Campaign services were also described during household visits in the baseline census.

In addition, as low adult male participation was observed in the 2011 CHC (with men making up 34% of adult participants), efforts were made to increase male participation in the 2012 CHC [9]. After soliciting ideas from village leaders to increase male participation, a targeted incentive of a free T-shirt was introduced for each adult male participant upon campaign completion. The T-shirt incentive for men was also promoted during the community sensitization activities.

### Baseline Census

In April 2012 a census was performed to enumerate the baseline population as the last national census in Uganda occurred in 2002. With the help of local leaders, census workers enumerated and collected basic demographics (age, sex, marital status and occupation) on all household members. Information on household members not present during a census visit was obtained from the head of household or other adults. Workers recorded household locations using handheld global positioning system (GPS) devices (Etrex Legend H Navigator, Garmin) and fingerprint biometric measurements (U.are.u 4500, Digital Persona) on all persons home during the census.

### 2012 Community Health Campaign

The campaign occurred over five days in May 2012 at four sites across the community. Well-known public gathering sites (a market place, a government council headquarters, and two primary schools) were chosen by the village leaders and investigators based on their convenience for villagers and to minimize transport costs and distance traveled reaching the campaigns. The campaign offered point-of-care (POC) HIV, hypertension and diabetes screening, tuberculosis (TB) symptom screening for all, and malaria testing for participants with self-reported fever, as previously described [9]. All participants screening HIV antibody positive underwent confirmatory testing with a POC rapid testing algorithm, followed by POC CD4+ T cell count testing (PIMA, Inverness Medical) [9]. They then received on-site post-test counseling, and were given an appointment to the local health center for HIV treatment. Finger-prick diagnostics eliminated the need for phlebotomy at the campaign.

### Statistical Methods

Residence in Kakyerere parish was defined by enumeration in the census. Self-report during the campaign questionnaire was also accepted to define parish residence, but only if the participant could name a household member identified in the census. Fingerprint biometric measures were used to verify resident identity upon campaign registration; if fingerprinting failed, name-based matching was used. The prevalence of each disease was estimated with the sample proportion, where the number of participants tested for each disease served as the denominator. Unadjusted analyses of variables affecting CHC attendance among adults residents were based on Pearson's Chi-squared test for proportions, Student's t-test for means and the Wilcoxon rank sum test for medians. For a set of *a priori* specified characteristics (sex, single marital status and in-person contact at the census), adjusted analyses were based on targeted minimum loss-based estimation (TMLE) [11]. Specifically, TMLE was used to estimate the relative risk of each variable of interest, after controlling for the other factors and age continuously. Unlike standard logistic regression, TMLE avoids parametric modeling assumptions and yields marginal (unconditional) estimates.

### Ethics statement

Verbal consent was obtained for all participants in order to maintain anonymity during HIV testing at the community health campaign, as written consent would have been the only name-linked identifier between a participant and his/her HIV test result. Campaign uptake was linked to the census data by a digital fingerprint biometric alone. Children from 13–17 years could provide verbal consent if a parent/guardian was not present, consistent with Uganda MOH policy [12]. Children <13 years could not participate without an assenting parent/guardian present. Verbal consent was documented by study staff for each study participant during the census and at entry into the campaign, and linked to an anonymous study identification number. The Makerere University School of Medicine Research and Ethics Committee, the Ugandan National Council on Science and Technology, and the UCSF Committee on Human Research approved the consent procedures and the study.

## Results

### Census

Over 12 days in April 2012, 18 census workers enumerated 6,343 parish residents (29 residents/day per worker). Based on population projections from the 2002 Ugandan Census, there were an estimated 6,400 Kakyerere parish residents in 2012 [13]. 4,247 (67%) residents were at home during the census, and 2,096 residents (33%) were enumerated but not at home. Of 4,247 residents at home during the census, 4,071 (96%) provided fingerprint biometric measures, and 176 (4%) declined. 3,149 (50%) of residents enumerated in the census were adults (>18 years), of whom 1,714 (54%) were female. Adults were home during census visits more often than children (2345/3149 [74%] vs. 1902/3194 [60%], respectively). Among adults, women were more likely to be home during census visits than men (1396/1714 [81%] vs. 949/1435 [66%]; p<0.001), and the median age was greater among residents at home (32 [IQR: 24–45] years) than residents not at home (25 [IQR: 20–38] years; p<0.001). Married, widowed and divorced/separated adults were also more likely to be home than single adults (1850/2262 [82%] vs. 493/880 [56%], respectively; p<0.001). During the health campaign, 501 persons not enumerated in the census reported residence in the community and could name a specific household member from the census, for a total parish population of 6,844 people.

### Community Health Campaign

**Figure 1:**
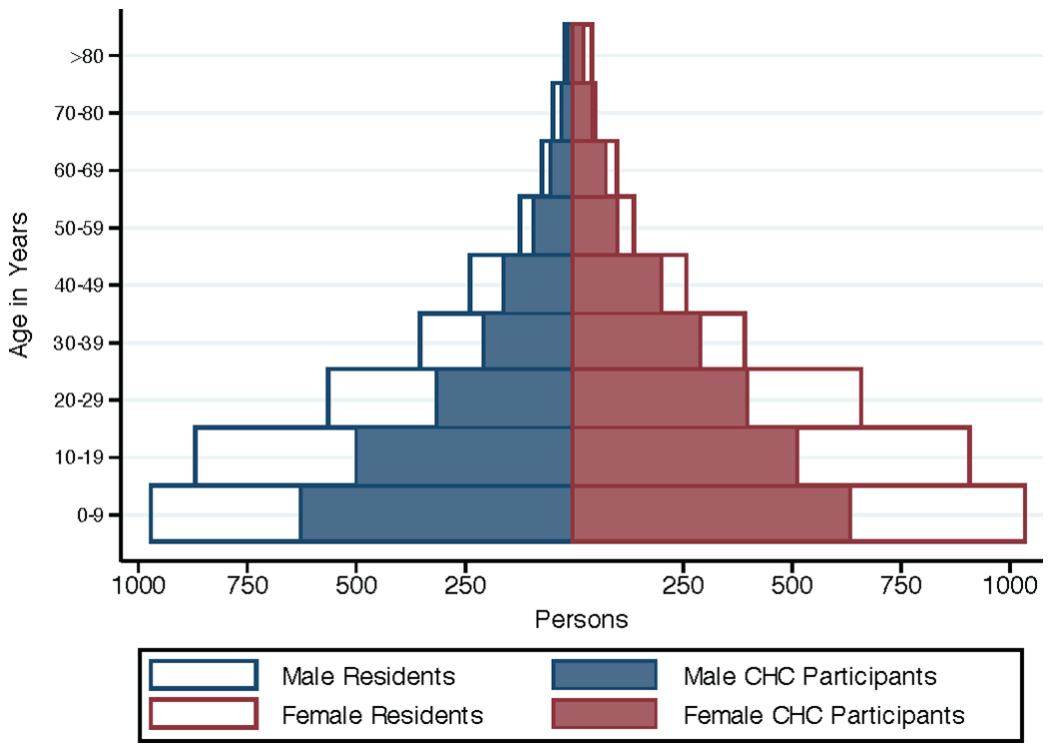
Population distribution of Kakyerere parish as determined from a twelve-day study census (open blue and red bars), and Community Health Campaign participation over five days among residents (solid blue and red bars), by age and sex.

**Figure 2:**
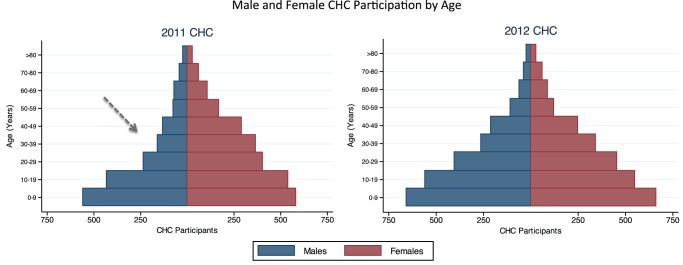
Change in male and female participation from a 2011 to a 2012 community health campaign (CHC) in Kakyerere parish, a rural Ugandan community. Shown are the age and sex distribution of CHC participants, including non-residents of the community, by year. The dashed arrow indicates the low proportion of adult male participants in the 2011 CHC.

In five days, 4,879 people participated in the health campaign for an average of 976 participants/day. 4,282 (63%) of 6,844 community residents participated (see Figure 1), and 597/4,879 (12%) participants were non-residents from neighboring communities. Overall, 55% (2,687/4,879) of participants were adults, and 1,249 (46%) adult participants were men (see Figure 2). HIV tests were performed in 4,795/4,879 (98.3%) participants, of whom 1,836 (38%) reported no prior HIV testing. Of 257 HIV-infected adults, 125 (49%) reported newly diagnosed HIV. Median CD4 count among newly diagnosed adults was 436 cells/μL and 90 (72%) had a CD4 cell count >350 cells/μL. Among HIV-infected adults, 199/257 (77%) had at least one of four TB symptoms recommended by the WHO for intensified TB case finding [14]. Among HIV-uninfected adults, 318/2,417 (13%) reported cough for >2 weeks. 1,234/4,879 (25%) of CHC participants reported fever, and 51 (4%) tested positive for malaria by RDT. The prevalence of hypertension and diabetes among adult participants was 18% and 2%, respectively. Screening results are shown in Table 1. Among adult residents participating in the campaign, 1,071/2,204 (51%) reported attending our CHC held in the same community in May 2011. The final campaign day was at the local trading center on a weekend market day. A significantly greater proportion of adults attending the trading center site, compared to the other three campaign sites, were male (55% vs. 44%; p<0.001), single (26% at trading center vs. 20% at other sites; p = 0.001), and younger (median age: 30 vs. 34 years; p<0.001).

**Table 1:** Screening results by disease during a five-day Community Health Campaign in Kakyerere parish, Uganda.

*Community Health Campaign Participants N = 4,879*	Number of participants screening positive	%
**HIV** (N = 4,795 tested)		
Children (<18 years) (N = 2,121)	12	1%
Adult (>18 years) (N = 2,674)	257	10%
Median CD4 count, adults (N = 210)	426 cells/μL	IQR: 306–613
New diagnoses in HIV-infected adults (N = 257)	125	49%
Median CD4 in newly diagnosed adults (N = 101)	436 cells/μL	IQR: 306–617
**TB symptoms, adults** (N = 2,674 screened)		
HIV-uninfected (N = 2,417)		
Cough >2 weeks	318	13%
HIV-infected (N = 257)		
Current cough	89	35%
Fever	81	32%
Weight loss	83	32%
Night sweats	125	49%
Any of above 4 symptoms	199	77%
**Malaria** (N = 4,879 screened for fever)		
Self-reported fever	1234	25%
Confirmed malaria, if febrile	51	4%
Age <10 years (N = 341)	27	8%
Age >10 years (N = 893)	24	3%
**Hypertension, adults** (N = 2,687 screened)		
Systolic>140 or diastolic>90 mmHg, or prior self-reported diagnosis	483	18%
**Diabetes, adults** (N = 2,672 screened)		
Adults with positive screening test	63	2%
New adult diagnoses	18	29%
**Smoking** (self-report)		
Men (N = 1246 screened)	393	32%
Women (N = 1437 screened)	152	11%

### Predictors of campaign participation

**Figure 3:**
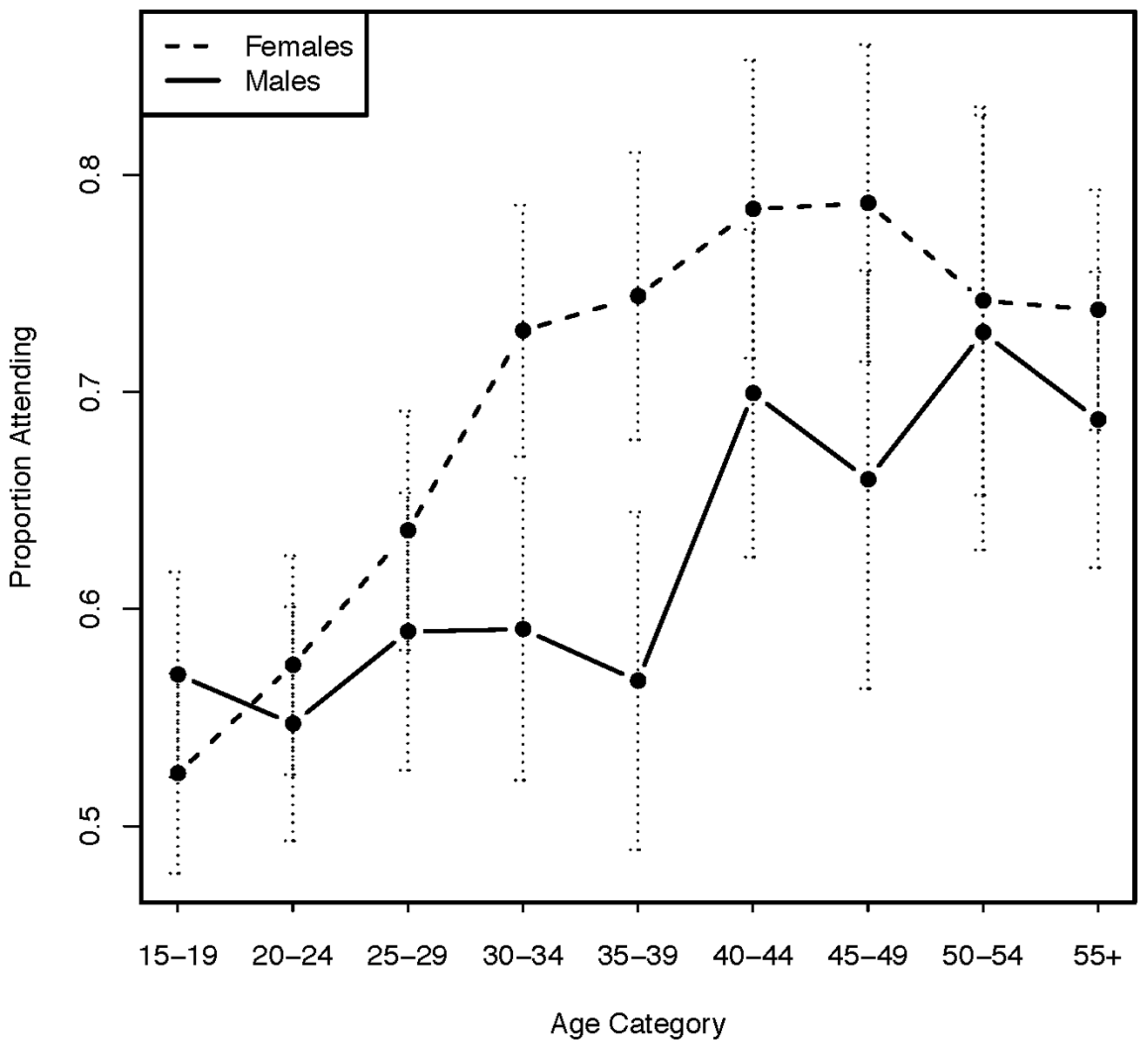
Proportion of residents attending the 2012 community health campaign (CHC) according to sex and age group among residents ≥15 years old. The dashed vertical lines indicate 95% confidence intervals.

Of 6,844 community residents, 4,282 (63%) residents participated and 2,562 (37%) residents did not participate in the campaign. Adult residents were more likely to participate than children (2,204/3,446 [64%] vs. 2,078/3,398 [61%], respectively; unadjusted p = 0.017). In unadjusted analyses, adult participants compared to non-participants were more likely to be female (1222/1843 [66%] women vs. 982/1,603 [61%] men participated; p = 0.002), older (median age: 32 [IQR: 24–45] years in participants vs. 27 [22–38] in non-participants; p<0.001), non-single (70% of married/widowed/divorced vs. 48% single adults participated; p<0.001), farmers (75% of farmers vs. 51% of non-farmers participated; p<0.001) and to have been home during the census (70% adults at home vs. 34% not at home participated; p<0.001). Figure 3 shows the proportion of each sex and age group that attended the campaign. The average distance from household to nearest campaign site was not significantly different in campaign participants (1.14 km) vs. non-participants (1.08 km; p = 0.87). Campaign participation by the head of household was significantly associated with campaign participation by other household members (68% [2,041/3,516] participation if household head participated vs. 41% [695/1,680] participation if household head did not participate; p<0.001).

After multivariate adjustment with TMLE, the estimated probability of attending the campaign was significantly lower among single compared to non-single adults (RR: 0.63; p<0.001 after adjusting for sex, being home during the census and age), and among adults who were not at home vs. at home during the census (adjusting for sex, single marital status, and age, RR: 0.83; p<0.001), whereas sex was no longer significantly associated with participation (Table 2).

**Table 2:** Results from the unadjusted and adjusted analyses of the *a priori*-specified variables of interest on the relative risk (RR) of CHC attendance.

Variable	Unadjusted RR	95% CI	p-value	Adjusted RR	95% CI	p-value
Male	0.92	0.89–0.98	0.002	0.99	0.94–1.04	0.76
Single	0.68	0.63–0.73	<0.001	0.63	0.53–0.74	<0.001
Contacted during census	1.33	1.25–1.41	<0.001	1.20	1.13–1.28	<0.001

The reference groups were female, married/widowed/divorced/separated and not-contacted, respectively. The adjusted analyses (TMLE) controlled for the other factors and age continuously.

## Discussion

Our findings demonstrate the continued yield of multi-disease, community health campaign (CHC)-based HIV testing in identifying persons with no prior testing and with undiagnosed HIV infection in a community with year-round access to facility-based testing. Using rigorous methods to define community residence, including a baseline census with fingerprint biometric measurements for identification, we found that young, single adults were significantly less likely than older, non-single adults to access HIV-testing via CHCs, suggesting that novel testing approaches are needed to reach this high-risk group.

Multi-disease CHCs have been successfully implemented in Uganda and Kenya and represent an effective and rapid strategy for scale-up of HIV testing services in rural, resource-limited settings [9], [10]. Although several modalities for non-facility based testing have been successfully implemented in rural Africa, including home-based testing (HBT) and mobile testing vans, CHCs offer the advantages of acting as a platform for multi-disease testing and rapid scale-up to population coverage in a period of days. With point-of-care CD4 count testing and co-location of HIV clinic staff at the CHC, the early cascade of HIV care (testing, counseling and disease staging) can be performed in a single encounter taking less than three hours. Multi-disease service provision may also encourage repeat HIV testing or testing on a regular basis, which is likely to be a necessary part of any “test and treat” approach, and indeed we found that half of the campaign participants reported HIV testing at a CHC held one year prior.

However, our findings also suggest that complementary approaches will be needed to achieve universal HIV testing across a population. By understanding how uptake varies with testing strategy, non-facility based testing strategies may be optimized to achieve universal testing coverage. Factors found to decrease uptake of home-based HIV testing (HBT) have varied considerably depending on the population studied, and include older age (>25 years) [4], as well as young adulthood [15], having a concurrent partnership at the time of HBT [4], lack of participation by the male head of household [16], single marital status, higher educational attainment [17], and high (>30%) prior rates of HIV testing in a community [18]. In our study population several factors were associated with lower testing uptake, notably younger age and single marital status. How these factors influence testing at CHCs remains unknown and multiple causal pathways are likely at play. For example, increased testing uptake with older age may be due to interest in the non-communicable disease screening offered at CHCs, whereas increased uptake among persons whose head of household participated in testing suggests that social networks influence CHC participation.

Low HIV testing uptake among young adults is a particularly important challenge due to the high HIV risk they face as they enter adulthood, and the enormous opportunity for HIV prevention among African youth. Despite the high risk of HIV faced by young adults, their testing rates are among the lowest of any demographic, particularly among young men, [19], [20], [21] and neither CHCs nor home-based testing approaches are immune to this [15]. A better understanding of the reasons for low HIV testing uptake among young, single adults and their perception of HIV risk is needed, and could inform novel approaches to reaching young adults and adolescents. Lower engagement by young adults in HIV prevention activities is not limited to HIV testing. Trials of HIV prevention interventions, such as pre-exposure prophylaxis with oral tenofovir-containing regimens have also found lower adherence as measured by drug levels among young women (age <25 years) and unmarried women in sub-Saharan Africa, further highlighting the challenges in HIV prevention interventions among young adults [22]. In spite of this challenge, community (non-facility) based HIV testing approaches such as CHCs have achieved higher testing uptake among young adults than health facility based testing and represent an opportunity to engage young adults in their communities for HIV prevention messaging and interventions. The relatively higher rate of young adult testing in the CHC seen at the marketplace also suggests that selecting testing sites with optimal convenience for young, working adults may increase testing uptake, and merits further exploration.

Despite lower rates of participation by young adults, adult male CHC participation was increased in the 2012 campaign to nearly half of adult participants (see Figure 2). Through a process of soliciting village leader input for a specific barrier to CHC uptake, incentives may have played a significant role in increasing male CHC participation, as has been described in a mobile testing approach in South Africa [23]. Several factors may have contributed to increased male participation in 2012 as well, such as increased familiarity with the campaign from the 2011 CHC, and inclusion of a marketplace as a campaign site. Lower HIV testing rates among men compared to women has been well described in a variety of settings, [9], [10], [21], [24] and likely contributes to lower rates of ART uptake in men [25], [26]. CHCs, incorporating input from local leadership, multi-disease services and simple incentives, offer a means of rapidly increasing adult male HIV testing uptake and reaching populations that might otherwise “fall through the cracks” of facility-based testing.

Our study has several limitations. The campaign was designed to accommodate 1,000 persons per day, and was limited to five days. The additional testing yield of having more campaign days, particularly among young, single adults, remains unknown. In addition, on the only campaign day occurring on a weekend, we observed a significantly younger median age and a greater proportion of single and male adults than the other four campaign days. This suggests that the lower participation rates of young, single adults may have been due in part to conflicts with their work schedules rather than lack of interest. The incentive used, a free T-shirt for adult males, was chosen by this community's leadership, and might not be effective or feasible in a different setting. Finally, our finding that being home during the census was significantly associated with campaign participation may reflect migration patterns, with persons not available during the census at higher likelihood of being away from home during the campaign days, rather than a mobilizing effect of the census. Despite these limitations, our data provide a rigorous evaluation of uptake of HIV testing using a CHC approach in a rural Ugandan community.

## Conclusions

In light of the increasing number of effective biomedical options for HIV prevention among both HIV-infected and uninfected persons, and increased access to antiretroviral therapy in sub-Saharan Africa, the opportunities for reducing HIV-associated morbidity and mortality and dramatically reducing HIV transmission are greater than ever, but depend on knowing one's HIV status. Achieving universal HIV testing across large populations will likely require several complementary testing approaches. Multi-disease community health campaigns provide a rapid means of jump-starting this process, and will serve as the engine driving universal HIV testing in the SEARCH “test and treat” cluster randomized-control trial we are conducting in East Africa, comparing universal HIV testing with antiretroviral therapy (ART) start at any CD4+ T cell count vs. CD4-guided ART start.

## Acknowledgments

We thank the residents of Kakyerere Parish, Mbarara District, Uganda, for their generous participation in our study. We also thank the village local councilors from Kakyerere Parish, the staff of the Bwizibwera Level IV Health Centre, Mbarara and the Mulago-Mbarara Joint AIDS Programme (MMJAP) for their assistance, and the Uganda Ministry of Health for the donation of HIV rapid test kits and male condoms for use in this health campaign.
